# Highly Pathogenic Avian Influenza A(H5N1) Virus Outbreak in New England Seals, United States

**DOI:** 10.3201/eid2904.221538

**Published:** 2023-04

**Authors:** Wendy Puryear, Kaitlin Sawatzki, Nichola Hill, Alexa Foss, Jonathon J. Stone, Lynda Doughty, Dominique Walk, Katie Gilbert, Maureen Murray, Elena Cox, Priya Patel, Zak Mertz, Stephanie Ellis, Jennifer Taylor, Deborah Fauquier, Ainsley Smith, Robert A. DiGiovanni, Adriana van de Guchte, Ana Silvia Gonzalez-Reiche, Zain Khalil, Harm van Bakel, Mia K. Torchetti, Kristina Lantz, Julianna B. Lenoch, Jonathan Runstadler

**Affiliations:** Tufts University Cummings School of Veterinary Medicine, North Grafton, Massachusetts, USA (W. Puryear, K. Sawatzki, A. Foss, J.J. Stone, M. Murray, E. Cox, J. Runstadler);; University of Massachusetts, Boston, Massachusetts, USA (N. Hill);; Marine Mammals of Maine, Brunswick, Maine, USA (L. Doughty, D. Walk, K. Gilbert);; New England Wildlife Centers, Barnstable, Massachusetts, USA (P. Patel, Z. Mertz);; New England Wildlife Centers, Weymouth, Massachusetts, USA (Z. Mertz);; Wild Care, Inc., Eastham, Massachusetts, USA (S. Ellis, J. Taylor);; National Oceanic and Atmospheric Administration Fisheries, Silver Spring, Maryland, USA (D. Fauquier);; National Oceanic and Atmospheric Administration Fisheries, Gloucester, Massachusetts, USA (A. Smith);; Atlantic Marine Conservation Society, Hampton Bays, New York, USA (R.A. DiGiovanni Jr.);; Mount Sinai Icahn School of Medicine, New York, New York, USA (A. van de Guchte, A.S. Gonzalez-Reiche, Z. Khalil, H. van Bakel);; US Department of Agriculture Animal and Plant Health Inspection Service, Ames, Iowa, USA (M.K. Torchetti, K. Lantz);; US Department of Agriculture Animal and Plant Health Inspection Service, Fort Collins, Colorado, USA (J.B. Lenoch)

**Keywords:** avian influenza, H5N1, influenza, viruses, zoonoses, virology, ecology, Caniformia, harbor seals, gray seals, New England, United States

## Abstract

We report the spillover of highly pathogenic avian influenza A(H5N1) into marine mammals in the northeastern United States, coincident with H5N1 in sympatric wild birds. Our data indicate monitoring both wild coastal birds and marine mammals will be critical to determine pandemic potential of influenza A viruses.

Highly pathogenic avian influenza (HPAI) viruses are of concern because of their pandemic potential, socioeconomic impact during agricultural outbreaks, and risks to wildlife conservation. Since October 2020, HPAI A(H5N1) virus, belonging to the goose/Guangdong H5 2.3.4.4b clade, has been responsible for >70 million poultry deaths and >100 discrete infections in many wild mesocarnivore species (*1*). As of January 2023, H5N1 infections in mammals have been primarily attributed to consuming infected prey, without evidence of further transmission among mammals.

We report an HPAI A(H5N1) virus outbreak among New England harbor and gray seals that was concurrent with a wave of avian infections in the region, resulting in a seal unusual mortality event (UME); evidence of mammal adaptation existed in a small subset of seals. Harbor (*Phoca* *vitulina*) and gray (*Halichoerus* *grypus*) seals in the North Atlantic are known to be affected by avian influenza A virus and have experienced previous outbreaks involving seal-to-seal transmission (*2*–*7*). Those seal species represent a pathway for adaptation of avian influenza A virus to mammal hosts that is a recurring event in nature and has implications for human health.

## The Study

**Figure 1:**
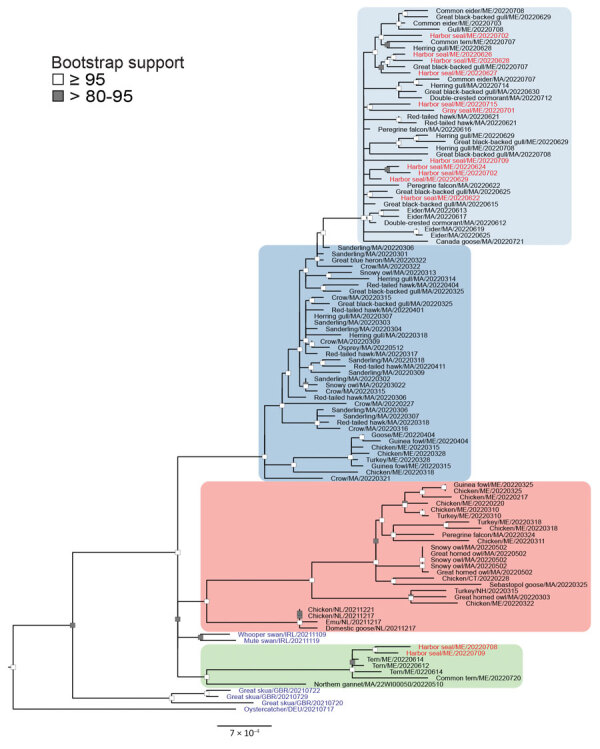
Phylogenetic analysis of highly pathogenic avian influenza A(H5N1) viruses from New England birds and seals, United States. Complete genomes of HPAI H5N1 viruses (GISAID database, https://www.gisaid.org) were compared by using IQ-TREE (https://www.iqtree.org) with the Ultrafast bootstrap (n = 10,000) option and A/chicken/NL/FAV-0033/2021 as a reference. Bootstrap support values >80 are shown at nodes. Red text indicates seal-derived sequences, black text avian-derived sequences from New England and Newfoundland, and blue text indicates avian-derived sequences from Europe . Branches are shaded on the basis of lineage groups: primary lineage from North America, pink; New England–specific lineage A, 1st wave blue, 2nd wave light blue; and New England–specific lineage B, green. All newly reported specimens were collected in the New England region during February–July 2022. Scale bar indicates nucleotide substitutions per site.

The first detections of HPAI clade 2.3.4.4b viruses in North America were in wild and domestic birds in November 2021 in Canada and late December 2021 in the United States (*8*–*11*). Starting on January 20, 2022, avian oropharyngeal or cloacal samples were collected from wild birds by personnel in 4 wildlife clinics in Massachusetts. Additional opportunistic samples were collected in Maine and Massachusetts in response to suspicious avian deaths in seabird breeding colonies. We screened samples from 1,079 individual wild birds representing 78 avian species of concern for H5 influenza and identified 119 infected birds from 21 species (Appendix 1 Figure 1, panel A; Appendix 2). Wild birds in New England experienced 2 waves of influenza infections during 2022. The first wave peaked in March and was largely represented by raptor deaths (39.1% influenza-positive birds). A second wave began in June; gull (38% influenza-positive) and eider (26.8% influenza-positive) deaths were most frequently reported during the second wave. Mortality events affected seabird breeding colonies throughout the coastal region during the second wave; 8 islands had >1 bird test positive for H5 (Appendix 1 Table).

During January 20–July 31, 2022, opportunistic nasal, oral, conjunctival or rectal swab samples were collected from 132 stranded seals along the North Atlantic coast from Maine to Virginia (Appendix 2). HPAI virus was not detected in any of the 82 seals that were sampled through May 31, 2022. Concurrent with the second wave of avian infections, increased seal strandings in Maine led to a National Oceanic and Atmospheric Administration declaration of a UME beginning on June 1, 2022, that included 164 harbor and 11 gray seals in Maine during June and July (*12*). Swab samples were collected from 41 of those animals; 17/35 harbor and 2/6 gray seals were HPAI-positive and were within coastal regions of known and suspected HPAI outbreaks among terns, eiders, cormorants, and gulls (Appendix 1 Figure 1, panel B). Respiratory symptoms were observed with a subset of neurologic cases, although most stranded seals were deceased. The respiratory tract was the most consistent source of reverse transcription PCR–positive samples from affected seals (15/19 nasal, 16/19 oral, 6/19 conjunctiva, and 4/19 rectal samples).

**Figure 2:**
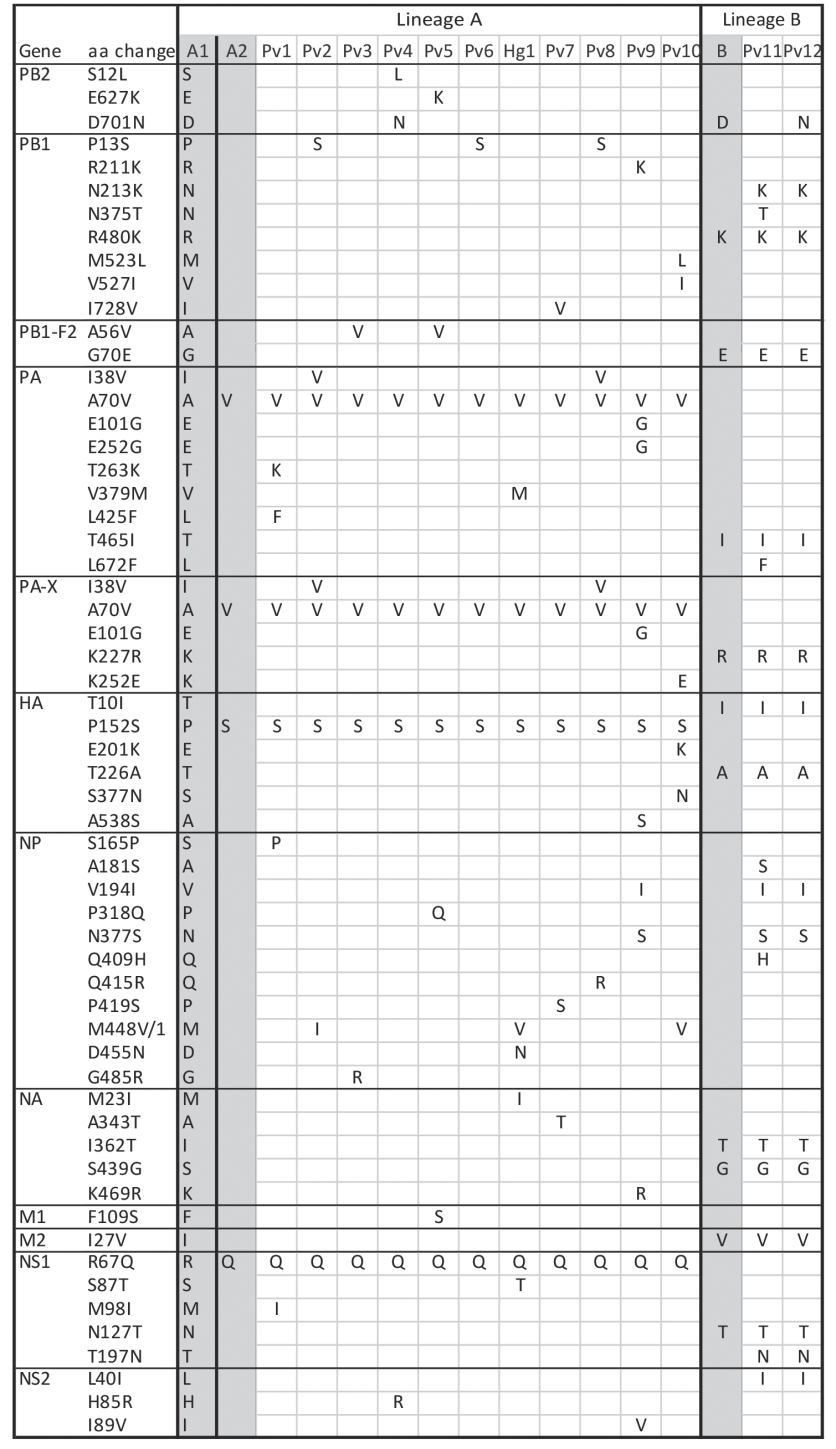
Amino acid changes in highly pathogenic avian influenza A(H5N1) viruses from New England birds and seals, United States. Each single-nucleotide polymorphism (SNP) that resulted in an amino acid change within ≥1 seal-derived sequence is shown. SNPs were observed in 12 H5N1 virus genes resulting in amino acid changes in corresponding proteins: PB2, PB1, PB1-F2, PA, PA-X, HA, NP, NA, M1, M2, NS1, and NS2. All avian virus reference sequences are shaded gray. A/Sanderling/MA/CW_22–112 (H5N1) (GISAID database, https://www.gisaid.org) (labeled A1) was used as a reference for first wave lineage A sequences; A/common eider/MA/TW_22–1400 (H5N1) (labeled A2) was used as a reference for second wave lineage A sequences; and A/common tern/MA/20220612_1 (H5N1) (labeled B) was used as a reference for lineage B sequences. Four aa differ between first and second wave lineage A viruses and 10 aa differ between first wave lineage A and lineage B. Second wave lineage A and lineage B seal-derived virus sequences, sampling date, and sampling location in Maine, USA, are indicated for each seal as follows: Pv1, MME22-112, 2022 Jun 22, Wells; Pv2, MME22-117, 2022 Jun 24, Yarmouth; Pv3, MME22-121, 2022 Jun 26, Georgetown; Pv4, MME22-122, 2022 Jun 27, New Harbor; Pv5, MME22-131, 2022 Jun 28, Harpswell; Pv6, MME22-133, 2022 Jun 29, S. Portland; Hg1, MME22-144, 2022 Jul 1, Phippsburg; Pv7, MME22-150, 2022 Jul 2, Westport; Pv8, MME22-155, 2022 Jul 2, Falmouth; Pv9, MME22-198, 2022 Jul 9, Wells; Pv10, MME22-230, 2022 Jul 15, Kennebunkport; Pv11, MME22-191, 2022 Jul 8, Harpswell; Pv12, MME22-195, 2022 Jul 9, Harpswell. HA, hemagglutinin; Hg, gray seal; M, matrix; NA, neuraminidase; NP, nucleoprotein; NS, nonstructural; PA, polymerase acidic; PB, polymerase basic; Pv, harbor seal.

We sequenced influenza A viruses from swab samples, resulting in 71 avian- and 13 seal-derived virus genomes from New England. We performed phylogenetic analysis of sequences from New England and the most closely related available virus sequences by using IQ-TREE (https://www.iqtree.org) (Figure 1; Appendix 1). We classified all but 1 virus as nonreassortant Eurasia 2.3.4.4b viruses and included those in further analyses (Appendix 1 Figures 2, 3). Sequences fell into 4 distinct clusters; 2 lineages were unique to New England. We found single-nucleotide polymorphisms (SNPs) (Appendix 1 Figures 4–7) and amino acid mutations (Figure 2) by using vSNP (https://github.com/USDA-VS/vSNP). Most sequences fell within a dominant New England–specific cluster that spanned the first and second waves (lineage A in this study). All second-wave viruses from lineage A exhibited the acquisition of new, shared mutations. That cluster spanned diverse species, including gulls, geese, eiders, raptors, and seals. A small number of raptor-derived sequences clustered with the primary lineage prevalent in North America at the time of sampling. All but 1 sample from the second wave of avian infections fell into either lineage A or a smaller, unique cluster primarily associated with terns (lineage B in this study).

We inferred that >2 spillover events occurred in the seal population during the second wave of avian infections. Of the sequences derived from seals, 11/13 clustered with second wave lineage A (Figure 1). We found 4 aa changes in specific proteins in both birds and seals that were distinct from the first wave of HPAI (polymerase acidic protein, A70V; polymerase acidic X protein, A62V; hemagglutinin protein, P152S; and nonstructural 1 protein, R67Q) (Figure 2). Within second wave lineage A, we found 37 aa changes in >1 seal sequence that were infrequent or absent from bird sequences. Most changes were unique; each occurred in only 1 animal. The polymerase basic 2 protein amino acid substitutions, E627K (in seal no. Pv/MME-22–131) and D701N (in seal no. Pv/MME-22–122), previously associated with mammalian adaptation were each present in 1 seal in second wave lineage A. An additional 2/13 seal-derived sequences clustered with lineage B; 10 aa mutations occurred in both bird and seal sequences (Figure 2). Another 10 aa changes occurred in at least 1 seal sequence that were infrequent or absent in the bird sequences. In contrast to lineage A, most amino acid changes were shared between the 2 seals in lineage B and were derived from animals stranded within the same town and sampled 1 day apart. The polymerase basic 2 protein substitution, D701N, was present in 1 seal from lineage B (Figure 2, seal no. PV12, MME-22–195).

## Conclusions

Transmission from wild birds to seals was evident for >2 distinct HPAI H5N1 lineages in this investigation and likely occurred through environmental transmission of shed virus. Viruses were not likely acquired by seals through predation or scavenging of infected animals, because birds are not a typical food source for harbor or gray seals (*13*). Data do not support seal-to-seal transmission as a primary route of infection. If individual bird–seal spillover events represent the primary transmission route, the associated seal UME suggests that transmission occurred frequently and had a low seal species barrier. We observed novel amino acid changes throughout the virus genome in seals, including amino acid substitutions associated with mammal adaptation.

In contrast to outbreaks in agricultural settings, outbreaks of HPAI in wild populations can rarely be managed well through biosecurity measures or depopulation, which is particularly true for large, mobile marine species such as seals. Avian and mammalian colonial wildlife might be particularly affected by influenza A viruses, which could enable ongoing circulation between and within species, providing opportunities for reassortments of novel strains and study of mammalian virus adaptation. Migratory animals might further disseminate the viruses over broad geographic regions. Therefore, the interface of wild coastal birds and marine mammals is critical for monitoring the pandemic potential of influenza A viruses.

Appendix 1 Additional information for highly pathogenic avian influenza A(H5N1) virus outbreak in New England seals, United States.

Appendix 2 Samples and sequences used for study of highly pathogenic avian influenza A(H5N1) virus outbreak in New England seals, United States.
