# Supplementary material for: Highly Pathogenic Avian Influenza A(H5N1) Virus Outbreak in New England Seals, United States
Source: Emerg Infect Dis. 2023 Apr;29(4):786–91. doi: 10.3201/eid2904.221538 (PMC10045683; doi:10.3201/eid2904.221538)
Supplement: Appendix 1 — Additional information for highly pathogenic avian influenza A(H5N1) virus outbreak in New England seals, United States. [file 22-1538-Techapp-s1.pdf]

Article DOI: https://doi.org/10.3201/eid2904.221538

EID cannot ensure accessibility for supplementary materials supplied by authors. Readers who have difficulty accessing supplementary content should contact the authors for assistance.

Highly Pathogenic Avian Influenza A(H5N1) Virus Outbreak in New England Seals, United States

Appendix 1

Materials and Methods

Sample Collection

Oropharyngeal or cloacal samples were collected from birds that were brought into wildlife clinics for care or were found deceased. Samples were collected from all live animals by experienced personnel within each facility (Tufts Wildlife Clinic, Cape Wildlife Clinic, New England Wildlife Centers, or Wild Care, Inc.) as part of diagnostic care. Deceased animals were either sampled in the field or brought to a wildlife center for sampling.

Oral, conjunctival, nasal, or rectal samples were collected from seals by staff at Marine Mammals of Maine, Seacoast Science Center, the National Marine Life Center, International Fund for Animal Welfare, Mystic Aquarium, and National Aquarium. Samples were obtained from both live and dead pinnipeds (gray, harbor, and harp seals) that were stranded along the Eastern Atlantic seaboard from Maine to Maryland during January–July 2022. Samples from stranded animals were collected as diagnostic samples under each organizations stranding agreement with the National Oceanic and Atmospheric Administration Fisheries Service.

Swab samples were collected by using polyester swabs (Puritan Medical Products, https://www.puritanmedproducts.com) that were placed into viral transport media (VTM) comprised of Medium 199, nystatin, gentamicin, benzylpenicillin, streptomycin, sulfamethoxazole, kanamycin sulfate (Sigma-Aldrich, https://www.sigmaaldrich.com) and bovine serum albumin (ThermoFisher Scientific, https://www.thermofisher.com). Samples were stored at −80°C or −20°C until processed.

Reverse Transcription PCR

RNA was extracted from 50 μL of VTM containing the sample by using the Mag-Bind Viral DNA/RNA 96 kit (Omega Bio-Tek Inc., https://www.omegabiotek.com) on a semiautomated KingFisher Purification System robot (ThermoFisher Scientific) as previously described (*7*). RNA was screened for the influenza A virus matrix protein (MP) gene by reverse transcription PCR (RT-PCR) by using the StepOnePlus platform (ThermoFisher Scientific). Total RNA (5 μL) was added to qScript XLT One-Step RT-qPCR ToughMix ROX (VWR, https://www.vwr.com) containing forward M F25 (5′-AGATGAGTCTTCTAACCGAGGTCG-3′) and reverse 2002 M R124 (5′-TGCAAAAACATCTTCAAGTCTCTG-3′) primers and M P64 (FAM-TCAGGCCCCCTCAAAGCCGA-TAM) probe. One-step real time RT-PCR was performed as follows: 50°C, 10 min; 95°C, 1 min; and 45 cycles of 95°C, 3 s and 60°C, 30 s. All plates were run with multiple negative VTM controls and purified A/Puerto Rico/8/1934 H1N1 RNA as a positive control. Any sample with a cycle threshold <40 was screened for the H5 hemagglutinin gene by using the same protocol described but with forward H5 1456NA (5′-ACGTATGACTATCCACCATACTCA-3′), forward H5 1456EA (5′-ACGTATGACTACCCGCAGTATTCA-3′), and reverse H5 1685 (5′-ACCTCGATGGGCAATGTGTT-3′) primers and H5 1637 (FAM-CATGTCCCTCATATCAAAACCTTCGGAGG-TAM) oligonucleotide probe. Samples with a cycle threshold <35 were sent to the US Department of Agriculture, National Veterinary Services Laboratories (NVSL, https://www.aphis.usda.gov) for further confirmatory RT-PCR testing.

Sequencing

Whole genome sequencing was performed at the Icahn School of Medicine at Mount Sinai, New York, USA, and NVSL. At the Icahn School of Medicine, original sample material from 25 animals was provided. RNA was extracted by using QIAmp Viral RNA minikits (QIAGEN, https://www.qiagen.com) and amplified by using a modified 2-step RT-PCR. RT was performed by using the ProtoScript II kit (New England Biolabs, https://www.neb.com) and 7 μL RNA and 1 μL of the Opti1 primer set. Reactions were incubated at 65°C for 5 min to denature the RNA, after which 10 μL of ProtoScript II Reaction Buffer and 2 μL of ProtoScript II Reverse Transcriptase were added; the reactions were then incubated at 25°C for 5 min, 48°C for 30 min, and 80°C for 5 min (to inactivate the enzyme). PCR was performed on cDNA by using a Q5 High-Fidelity PCR kit (New England Biolabs) in accordance with the manufacturer's

instructions; reactions were adjusted to a 25 µL volume containing 5 µL cDNA and the Opti1 primer set, consisting of Opti1-F1 (5′-GTTACGCGCCAGCAAAAGCAGG-3′), Opti1-F2 (5′-GTTACGCGCCAGCGAAAGCAGG-3′), and Opti1-R1 (5′-GTTACGCGCCAGTAGAAACAAGG-3′) primers. DNA amplicons were purified by using an Agencourt AMPure XP 5 mL kit (Beckman Coulter, https://www.beckman.com) and prepared by using the Nextera XT DNA Library Preparation kit (Illumina, https://www. illumina.com) following the manufacturer's protocol; sequencing was performed on a MiSeq instrument (Illumina) with 2 × 150-bp end reads. At the NVSL, original sample material from 38 animals was PCR-amplified and cDNA libraries were prepared by using the Nextera XT DNA Sample Preparation Kit (New England Biolabs) following the manufacturer's instructions. Sequencing was performed by using Reagent Kit v2 (500-cycles) on the MiSeq platform.

Phylogenetic analysis

Representative viruses obtained from a BLAST (https://www.ncbi.nlm.nih.gov/BLAST/) search were most closely related to viruses originating in Europe and North America but not to viruses from Africa or Asian. Therefore, we downloaded all available H5 influenza genome sequences from Europe (July 1, 2021–September 1, 2022) and North America (December 17, 2021–June 29, 2022) from the GISAID database (https://www.gisaid.org, accessed September 1, 2022). All virus segments were aligned with Clustal Omega v1.2.4, trimmed to the coding sequence, then concatenated to single supergenes. Low quality sequences were removed. Maximum-likelihood phylogenetic trees were estimated by using IQ-TREE v.2.1.3 with model selection on the total viral sample (n = 1,311) (Appendix Figure 2), a subset of all viruses from North America and the most closely related viruses from Europe (n = 407) (Appendix Figure 3), and a subset of only viruses from New England and Maritimes and the most closely related viruses from Europe (n = 107). The smallest subset was further analyzed by using the ultrafast bootstrap (n = 10,000) branch support and re-rooted by using TreeTime v0.9.4. One New England-derived virus had evidence of recombination (great black-backed gull/MA/22HP00488/20220723) and was excluded from the final subset. Comparative single nucleotide polymorphism (SNP) analysis was performed by using the NVSL vSNP pipeline (https://github.com/USDA-VS/vSNP) and Chicken/NL/FAV-0033/20221221 as a reference.

Appendix Table. New England seabird breeding colonies where suspicious avian deaths were observed*

Breeding colony locations	Town, state	GPS coordinates	Deaths	HPAI
Ship Island	Steuben, Maine	44.4334, −67.89743	Eiders	Confirmed
Green Island	Steuben, Maine	44.37341, −67.87305	Gulls, eiders, cormorants	Confirmed
Petit Manan Island	Steuben, Maine	44.3673, −67.86527	Gulls, eiders	Confirmed
Swans Island	Swans Island, Maine	44.18795, −68.44547	Gulls	Confirmed
Great Duck Island	Frenchboro, Maine	, 44.155, −68.24985	Gulls	Suspected
Mount Desert Rock	Mt Desert Rock, Maine	43.96869, −68.12778	Gulls	Suspected
Metinic Island	Vinalhaven, Maine	43.88708, −69.12581	Gulls, terns	Confirmed
Pond Island NWR	Phippsburg, Maine	43.73952, −69.77064	Terns	Confirmed
Appledore Island	Appledore Island, Maine	42.98678, −70.61424	Gulls	Suspected
Thacher Island NWR	Rockport, Massachusetts	42.63904, −70.57491	Gulls	Confirmed
Straitsmouth Island	Rockport, Massachusetts	42.65981, −70.59115	Eiders	Confirmed

*GPS, global positioning system; HPAI, highly pathogenic avian influenza.

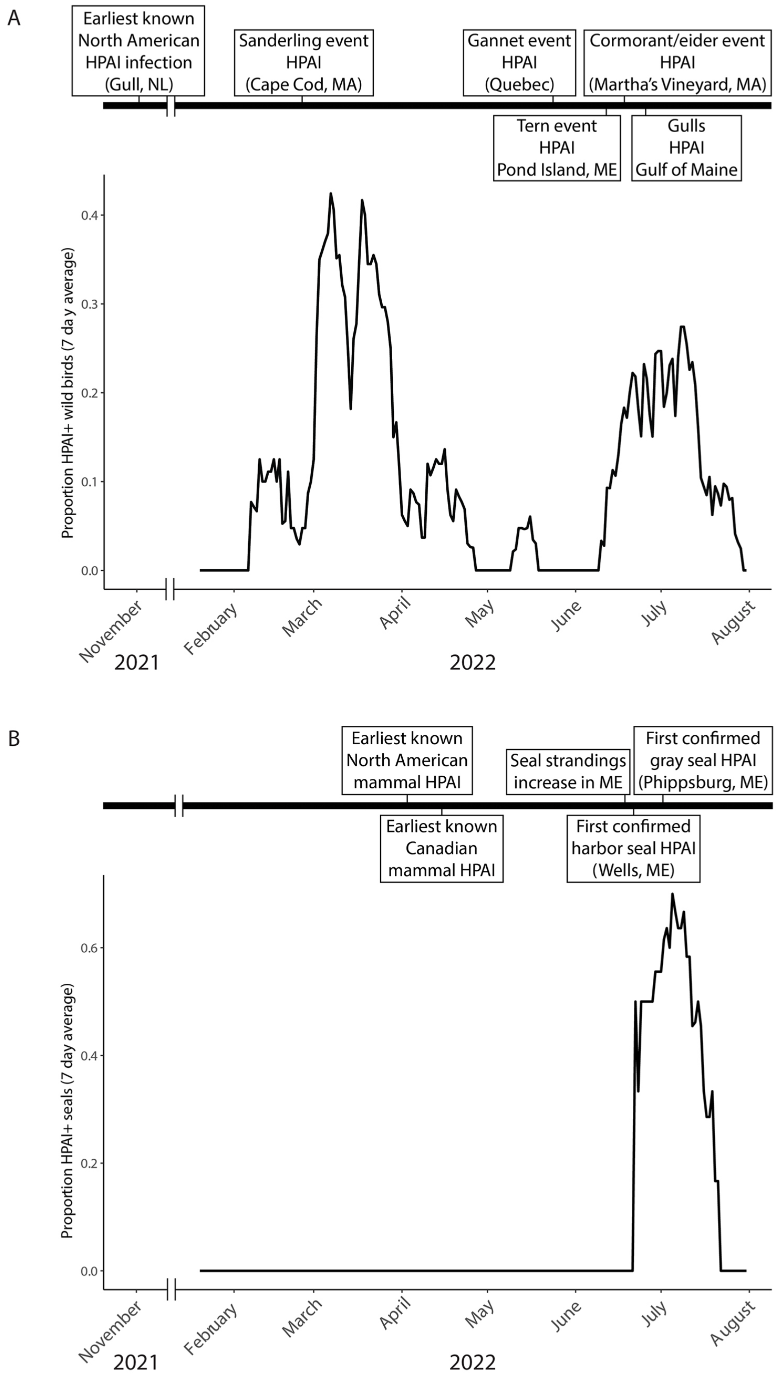

Appendix Figure 1. Opportunistic surveillance of New England birds and seals for highly pathogenic avian influenza (HPAI) H5N1 virus. Detection of HPAI in New England began in February 2022. A) Rolling 7-day average of H5-positive birds detected by reverse transcription PCR from New England wildlife clinics and opportunistic field collection (n = 1,079 unique birds). Timeline shows concurrent avian mortality events. Two waves of HPAI were observed in the region between February 2021 and August 2022. B) Rolling 7-day average of H5-positive seals stranded along the US Atlantic coast detected by reverse transcription PCR (n = 132 unique seals).

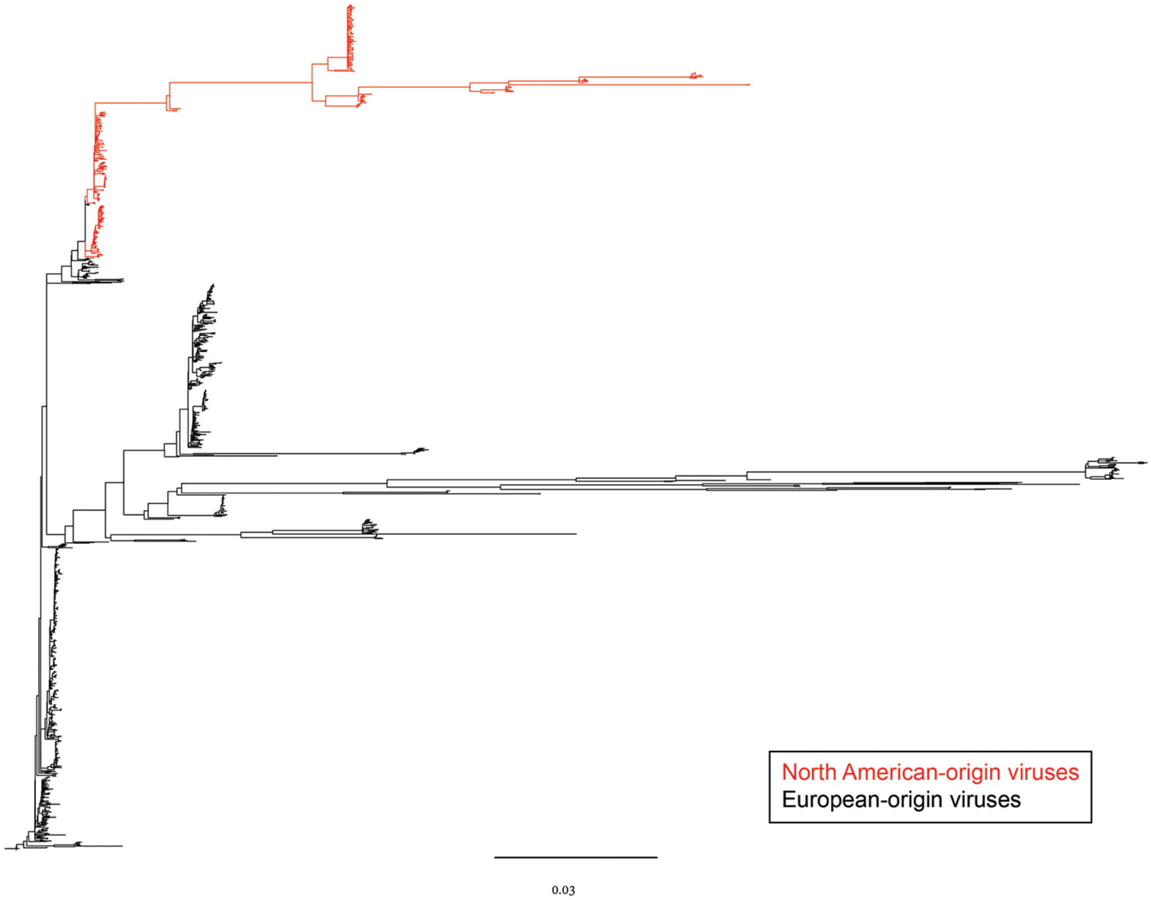

Appendix Figure 2. Phylogenetic analysis of H5N1 influenza viruses originating in North America and Europe. All available high-quality H5 influenza genome sequences from North America and Europe were collected, aligned, and trimmed to the coding sequence (n = 1,311). Gene segments were concatenated and maximum-likelihood phylogenetic trees were estimated by using IQ-TREE v.2.1.3 (https://www.iqtree.org) with model selection. Branches for Europe are shown in black and in red for North America. Scale bar indicates nucleotide substitutions per site.

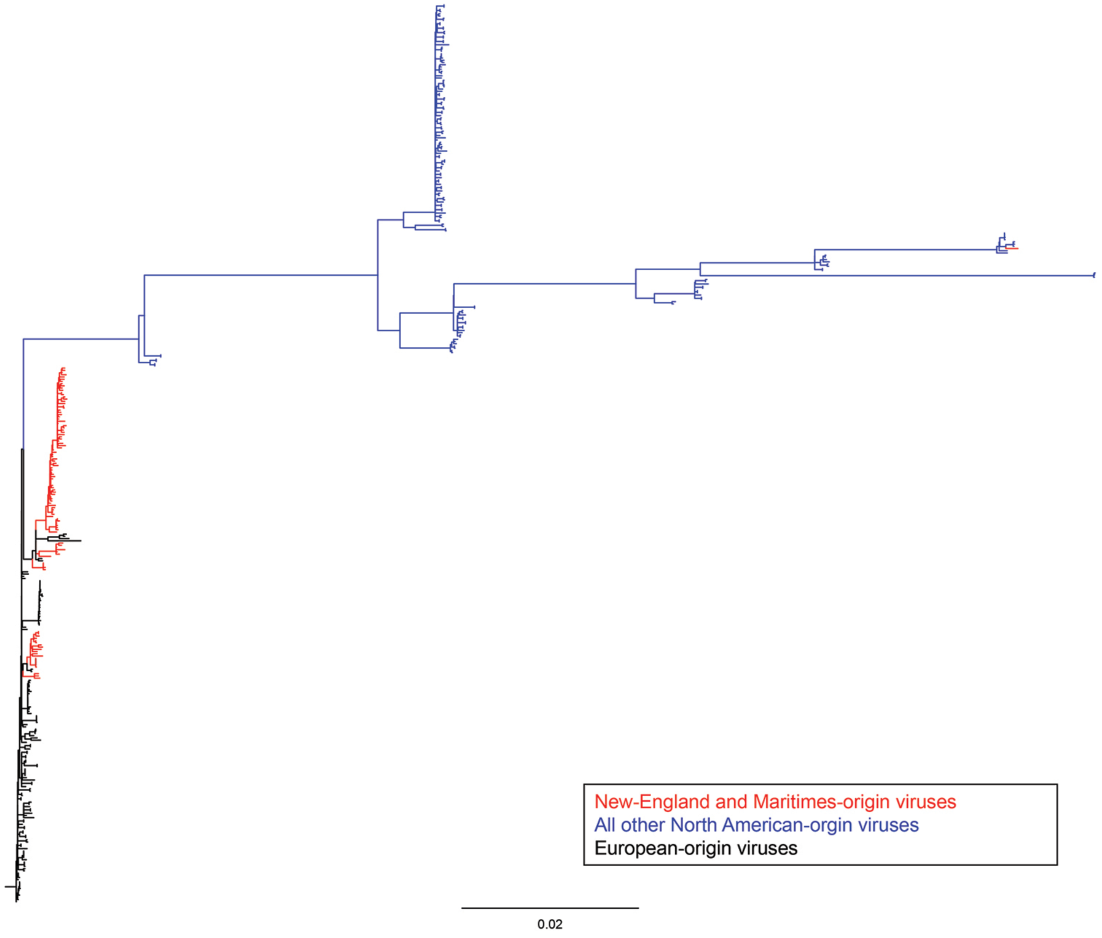

Appendix Figure 3. Phylogenetic analysis of H5N1 influenza viruses from New England and North America. All available high-quality H5 influenza genome sequences from North America and closely related Europe-derived sequences (as inferred in Appendix Figure 1) were aligned and trimmed to coding sequence (n = 407). Gene segments were concatenated and maximum-likelihood phylogenetic trees were estimated using IQ-TREE v.2.1.3 (https://www.iqtree.org) with model selection. Branches for viruses from New England and Maritimes are shown in red, all other viruses from North America are shown in blue, and branches for Europe are shown in black. Only 1 virus from New England had evidence of reassortment (great black-backed gull/MA/22HP00488/20220723, top right in tree) and was excluded from the final subset. Scale bar indicates nucleotide substitutions per site.

	PB2:39	PB2:321	PB2:555	PB2:1788	PB1:507	PB1:750	PB1:1362	PB1:1524	PB1:1836	PB1:2091	PB1:2100	PB1:2221	PA:666	PA:752	PA:1184	HA:300	HA:897	HA:969	HA:1032	HA:1194	HA:1263	HA:1405	NP:138	NP:465	NP:570	NP:816	NA:720	NS:592	NS:717	PB2:1490	PB2:1578	PB1:201	PB1:366	PA:147	NA:1097	MP:963	NS:835
Chicken/NL/FAV-0033/2021 reference	G	T	T	A	T	A	G	G	G	G	C	G	C	A	G	T	A	G	A	C	G	C	G	G	G	T	T	C	A	G	G	C	A	G	G	T	T
Peregrine falcon/MA/22MM00242/20220324	A	C	C	G	C	T	T	A	A	A	T	T	T	G	A	C	T	A	G	T	A	T	A	A	A	C	C	T	G	·	·	·	·	·	·	·	·
Great horned owl/MA/22MM00468/20220420	A	C	C	G	C	T	T	A	A	A	T	T	T	G	A	C	T	A	G	T	A	T	A	A	A	C	C	T	G	A	A	T	G	A	A	C	C
Gray seal/ME/20220701	·	·	·	·	·	·	·	·	·	·	·	·	·	·	·	·	·	·	·	·	·	·	·	·	·	·	·	·	·	·	·	·	·	·	·	·	·
Harbor seal/ME/20220709	·	·	·	·	·	·	·	·	·	·	·	·	·	·	·	·	·	·	·	·	·	·	·	·	·	·	·	·	·	·	·	·	·	·	·	·	·
Harbor seal/ME/20220715	·	·	·	·	·	·	·	·	·	·	·	·	·	·	·	·	·	·	·	·	·	·	·	·	·	·	·	·	·	·	·	·	·	·	·	·	·
Peregrine falcon/MA/20220616	·	·	·	·	·	·	·	·	·	·	·	·	·	·	·	·	·	·	·	·	·	·	·	·	·	·	·	·	·	·	·	·	·	·	·	·	·
Common eider/MA/20220707	·	·	·	·	·	·	·	·	·	·	·	·	·	·	·	·	·	·	·	·	·	·	·	·	·	·	·	·	·	·	·	·	·	·	·	·	·
Great black-backed gull/MA/20220625	·	·	·	·	·	·	·	·	·	·	·	·	·	·	·	·	·	·	·	·	·	·	·	·	·	·	·	·	·	·	·	·	·	·	·	·	·
Harbor seal/ME/20220622	·	·	·	·	·	·	·	·	·	·	·	·	·	·	·	·	·	·	·	·	·	·	·	·	·	·	·	·	·	·	·	·	·	·	·	·	·
Harbor seal/ME/20220629	·	·	·	·	·	·	·	·	·	·	·	·	·	·	·	·	·	·	·	·	·	·	·	·	·	·	·	·	·	·	·	·	·	·	·	·	·
Harbor seal/ME/20220624	·	·	·	·	·	·	·	·	·	·	·	·	·	·	·	·	·	·	·	·	·	·	·	·	·	·	·	·	·	·	·	·	·	·	·	·	·
Peregrine falcon/MA/20220622	·	·	·	·	·	·	·	·	·	·	·	·	·	·	·	·	·	·	·	·	·	·	·	·	·	·	·	·	·	·	·	·	·	·	·	·	·
Harbor_seal/ME/20220702	·	·	·	·	·	·	·	·	·	·	·	·	·	·	·	·	·	·	·	·	·	·	·	·	·	·	·	·	·	·	·	·	·	·	·	·	·
Harbor seal/ME/20220627	·	·	·	·	·	·	·	·	·	·	·	·	·	·	·	·	·	·	·	·	·	·	·	·	·	·	·	·	·	·	·	·	·	·	·	·	·
Great black-backed gull/ME/20220707	·	·	·	·	·	·	·	·	·	·	·	·	·	·	·	·	·	·	·	·	·	·	·	·	·	·	·	·	·	·	·	·	·	·	·	·	·
Harbor seal/ME/20220628	·	·	·	·	·	·	·	·	·	·	·	·	·	·	·	·	·	·	·	·	·	·	·	·	·	·	·	·	·	·	·	·	·	·	·	·	·
Harbor seal/ME/20220626	·	·	·	·	·	·	·	·	·	·	·	·	·	·	·	·	·	·	·	·	·	·	·	·	·	·	·	·	·	·	·	·	·	·	·	·	·
Harbor seal/ME/20220702	·	·	·	·	·	·	·	·	·	·	·	·	·	·	·	·	·	·	·	·	·	·	·	·	·	·	·	·	·	·	·	·	·	·	·	·	·
Herring gull/ME/20220628	·	·	·	·	·	·	·	·	·	·	·	·	·	·	·	·	·	·	·	·	·	·	·	·	·	·	·	·	·	·	·	·	·	·	·	·	·
Common eider/ME/20220703	·	·	·	·	·	·	·	·	·	·	·	·	·	·	·	·	·	·	·	·	·	·	·	·	·	·	·	·	·	·	·	·	·	·	·	·	·
Great black-backed gull/ME/20220629	·	·	·	·	·	·	·	·	·	·	·	·	·	·	·	·	·	·	·	·	·	·	·	·	·	·	·	·	·	·	·	·	·	·	·	·	·
Herring gull/ME/20220629	·	·	·	·	·	·	·	·	·	·	·	·	·	·	·	·	·	·	·	·	·	·	·	·	·	·	·	·	·	·	·	·	·	·	·	·	·
Great black-backed gull/MA/20220708	·	·	·	·	·	·	·	·	·	·	·	·	·	·	·	·	·	·	·	·	·	·	·	·	·	·	·	·	·	·	·	·	·	·	·	·	·
Double-crested cormorant/MA/20220612	·	·	·	·	·	·	·	·	·	·	·	·	·	·	·	·	·	·	·	·	·	·	·	·	·	·	·	·	·	·	·	·	·	·	·	·	·
Canada goose/MA/20220721	·	·	·	·	·	·	·	·	·	·	·	·	·	·	·	·	·	·	·	·	·	·	·	·	·	·	·	·	·	·	·	·	·	·	·	·	·
Sanderling/MA/20220306	·	·	·	·	·	·	·	·	·	·	·	·	·	·	·	·	·	·	·	·	·	·	·	·	·	·	·	·	·	·	·	·	·	·	·	·	·
Sanderling/MA/20220301	·	·	·	·	·	·	·	·	·	·	·	·	·	·	·	·	·	·	·	·	·	·	·	·	·	·	·	·	·	·	·	·	·	·	·	·	·
Snowy owl/MA/20220313	·	·	·	·	·	·	·	·	·	·	·	·	·	·	·	·	·	·	·	·	·	·	·	·	·	·	·	·	·	·	·	·	·	·	·	·	·
Great black-backed gull/MA/20220325	·	·	·	·	·	·	·	·	·	·	·	·	·	·	·	·	·	·	·	·	·	·	·	·	·	·	·	·	·	·	·	·	·	·	·	·	·
Red-tailed hawk/MA/20220411	·	·	·	·	·	·	·	·	·	·	·	·	·	·	·	·	·	·	·	·	·	·	·	·	·	·	·	·	·	·	·	·	·	·	·	·	·
Sanderling/MA/20220302	·	·	·	·	·	·	·	·	·	·	·	·	·	·	·	·	·	·	·	·	·	·	·	·	·	·	·	·	·	·	·	·	·	·	·	·	·
Red-tailed hawk/MA/20220317	·	·	·	·	·	·	·	·	·	·	·	·	·	·	·	·	·	·	·	·	·	·	·	·	·	·	·	·	·	·	·	·	·	·	·	·	·
Red-tailed hawk/MA/20220306	·	·	·	·	·	·	·	·	·	·	·	·	·	·	·	·	·	·	·	·	·	·	·	·	·	·	·	·	·	·	·	·	·	·	·	·	·
Crow/MA/20220227	·	·	·	·	·	·	·	·	·	·	·	·	·	·	·	·	·	·	·	·	·	·	·	·	·	·	·	·	·	·	·	·	·	·	·	·	·
Crow/MA/20220321	·	·	·	·	·	·	·	·	·	·	·	·	·	·	·	·	·	·	·	·	·	·	·	·	·	·	·	·	·	·	·	·	·	·	·	·	·
Northern gannet/MA/20220510	·	·	·	·	·	·	·	·	·	·	·	·	·	·	·	·	·	·	·	·	·	·	·	·	·	·	·	·	·	·	·	·	·	·	·	·	·
Common tern/ME/20220720	·	·	·	·	·	·	·	·	·	·	·	·	·	·	·	·	·	·	·	·	A	·	·	·	·	·	·	·	·	·	·	·	·	·	·	·	·
Tern/ME/20220612	·	·	·	·	·	·	·	·	·	·	·	·	·	·	·	·	·	·	·	·	·	·	·	·	·	·	·	·	·	·	·	·	·	·	·	·	·
Harbor seal/ME/20220708	·	·	·	·	·	·	·	·	·	·	·	·	·	·	·	·	·	·	·	·	·	·	·	·	·	·	·	·	·	·	·	·	·	·	·	·	·
Harbor seal/ME/20220709	·	·	·	·	·	·	·	·	·	·	·	·	·	·	·	·	·	·	·	·	·	·	·	·	·	·	·	·	·	·	·	·	·	·	·	·	·

Primary North American lineage

2nd wave

New England-lineage a

1st wave

New England lineage b

Appendix Figure 4. Single nucleotide polymorphism pattern for primary H5N1 lineage from North America. Eighty-four H5N1 sequences collected from birds and seals in New England were processed by using the vSNP pipeline (https://github.com/USDA-VS/vSNP) and Chicken/NL/FAV-0033/2021 (GISAID database, https://www.gisaid.org) as a reference. Avian samples with redundant single nucleotide polymorphism (SNP) motifs were removed, and the earliest observed member was retained. All SNPs observed in a single bird were removed. All seal SNPs were retained. Seal viruses in lineage A are highlighted in red, and seal viruses in lineage B are highlighted in blue. The SNP motif associated with the primary North American lineage is shown. Complete SNP data are available in Appendix 2 (https://wwwnc.cdc.gov/EID/article/29/4/22-1538-App2.xlsx).

	PB2:583	PB2:1842	PA:1746	NP:150	NA:462	PB2:2067	PB1:852	PB1:1167	PB1:1439	PA:1394	HA:29	HA:447	HA:676	HA:972	NP:1130	NA:375	NA:379	NA:399	NA:669	MP:767	PB2:285	PB1:1611	NA:1224	NP:580	HA:1719
Chicken/NL/FAV-0033/2021 reference	A	A	G	A	T	G	T	C	A	T	T	A	G	A	G	C	T	C	T	G	T	T	G	A	A
Peregrine falcon/MA/22MM00242/20220324	·	·	·	·	·	·	·	·	·	·	·	·	·	·	·	·	·	·	·	·	·	·	·	·	·
Great horned owl/MA/22MM00468/20220420	·	·	·	·	·	·	·	·	·	·	·	·	·	·	·	·	·	·	·	·	·	·	·	·	·
Gray seal/ME/20220701	G	G	A	C	C	A	C	A	G	C	C	G	A	G	A	T	C	T	A	A	A	C	A	G	G
Harbor seal/ME/20220709	G	G	A	C	C	A	C	A	G	C	C	G	A	G	A	T	C	T	A	A	A	C	A	G	G
Harbor seal/ME/20220715	G	G	A	C	C	A	C	A	G	C	C	G	A	G	A	T	C	T	A	A	A	C	A	G	G
Peregrine falcon/MA/20220616	G	G	A	C	C	A	C	A	G	C	C	G	A	G	A	T	C	T	A	A	A	C	A	G	G
Common eider/MA/20220707	G	G	A	C	C	A	C	A	G	C	C	G	A	G	A	T	C	T	A	A	A	C	A	G	G
Great black-backed gull/MA/20220625	G	G	A	C	C	A	C	A	G	C	C	G	A	G	A	T	C	T	A	A	A	C	A	G	G
Harbor seal/ME/20220622	G	G	A	C	C	A	C	A	G	C	C	G	A	G	A	T	C	T	A	A	A	C	A	G	G
Harbor seal/ME/20220629	G	G	A	C	C	A	C	A	G	C	C	G	A	G	A	T	C	T	A	A	A	C	A	G	G
Harbor seal/ME/20220624	G	G	A	C	C	A	C	A	G	C	C	G	A	G	A	T	C	T	A	A	A	C	A	G	G
Peregrine falcon/MA/20220622	G	G	A	C	C	A	C	A	G	C	C	G	A	G	A	T	C	T	A	A	A	C	A	G	G
Harbor_seal/ME/20220702	G	G	A	C	C	A	C	A	G	C	C	G	A	G	A	T	C	T	A	A	A	C	A	G	G
Harbor seal/ME/20220627	G	G	A	C	C	A	C	A	G	C	C	G	A	G	A	T	C	T	A	A	A	C	A	G	G
Great black-backed gull/ME/20220707	G	G	A	C	C	A	C	A	G	C	C	G	A	G	A	T	C	T	A	A	A	C	A	G	G
Harbor seal/ME/20220628	G	G	A	C	C	A	C	A	G	C	C	G	A	G	A	T	C	T	A	A	A	C	A	G	G
Harbor seal/ME/20220626	G	G	A	C	C	A	C	A	G	C	C	G	A	G	A	T	C	T	A	A	A	C	A	G	G
Harbor seal/ME/20220702	G	G	A	C	C	A	C	A	G	C	C	G	A	G	A	T	C	T	A	A	A	C	A	G	G
Herring gull/ME/20220628	G	G	A	C	C	A	C	A	G	C	C	G	A	G	A	T	C	T	A	A	A	C	A	G	G
Common eider/ME/20220703	G	G	A	C	C	A	C	A	G	C	C	G	A	G	A	T	C	T	A	A	A	C	A	G	G
Great black-backed gull/ME/20220629	G	G	A	C	C	A	C	A	G	C	C	G	A	G	A	T	C	T	A	A	A	C	A	G	G
Herring gull/ME/20220629	G	G	A	C	C	A	C	A	G	C	C	G	A	G	A	T	C	T	A	A	A	C	A	G	·
Great black-backed gull/MA/20220708	G	G	A	C	C	A	C	A	G	C	C	G	A	G	A	T	C	T	A	A	A	C	A	G	G
Double-crested cormorant/MA/20220612	G	G	A	C	C	A	C	A	G	C	C	G	A	G	A	T	C	T	A	A	A	C	A	G	G
Canada goose/MA/20220721	G	G	A	C	C	A	C	A	G	C	C	G	A	G	A	T	C	T	A	A	A	C	A	G	G
Sanderling/MA/20220306	G	G	A	C	C	A	C	A	G	C	C	G	A	G	A	T	C	T	A	A	A	C	A	G	G
Sanderling/MA/20220301	G	G	A	C	C	A	C	A	G	C	C	G	A	G	A	T	C	T	A	A	A	C	A	G	G
Snowy owl/MA/20220313	G	G	A	C	C	A	C	A	G	C	C	G	A	G	A	T	C	T	A	A	A	C	A	G	G
Great black-backed gull/MA/20220325	G	G	A	C	C	A	C	A	G	C	C	G	A	G	A	·	C	T	A	A	A	C	A	G	G
Red-tailed hawk/MA/20220411	G	G	A	C	C	A	C	A	G	C	C	G	A	G	A	T	C	T	A	A	A	C	A	G	G
Sanderling/MA/20220302	G	G	A	C	C	A	C	A	G	C	C	G	A	G	A	T	C	T	A	A	A	C	A	G	G
Red-tailed hawk/MA/20220317	G	G	A	C	C	·	C	A	G	C	C	G	A	G	A	T	C	T	A	A	A	C	A	G	G
Red-tailed hawk/MA/20220306	G	G	A	C	C	A	C	A	G	C	C	G	A	G	A	T	C	T	A	A	A	C	A	G	·
Crow/MA/20220227	G	G	A	C	C	A	C	A	G	C	C	G	A	G	A	T	C	T	A	A	A	C	A	·	·
Crow/MA/20220321	G	G	A	C	C	A	C	A	G	C	C	G	A	G	A	T	C	T	A	A	·	·	·	·	·
Northern gannet/MA/20220510	G	G	A	C	C	·	·	·	·	·	·	·	·	·	·	·	·	·	·	·	·	·	·	·	·
Common tern/ME/20220720	G	G	A	C	C	·	·	·	·	·	·	·	·	·	·	·	·	·	·	·	·	·	·	·	·
Tern/ME/20220612	G	G	A	C	C	·	·	·	·	·	·	·	·	·	·	·	·	·	·	·	·	·	·	·	·
Harbor seal/ME/20220708	G	G	A	C	C	·	·	·	·	·	·	·	·	·	·	·	·	·	·	·	·	·	·	·	·
Harbor seal/ME/20220709	G	G	A	C	C	·	·	·	·	·	·	·	·	·	·	·	·	·	·	·	·	·	·	·	·

	NA:1085	MP:123	NP:171	PB2:351	HA:552	PA:209	PB1:756	PB1:2199	PA:1945	HA:454	HA:708	NS:200	PA:2031	PB2:0	PB1:37	NS:0	PA:1293	NS:294	MP:558	PB1:2182	PA:1275	NS:731	PA:78	PB2:1482	PB1:261
Chicken/NL/FAV-0033/2021 reference	C	T	C	C	T	C	G	A	C	C	C	G	A	T	C	A	C	G	T	A	T	T	A	A	C
Peregrine falcon/MA/22MM00242/20220324	·	·	·	·	·	·	·	·	·	·	·	·	·	·	·	·	·	·	·	·	·	·	·	·	·
Great horned owl/MA/22MM00468/20220420	·	·	·	·	·	·	·	·	·	·	·	·	·	·	·	·	·	·	·	·	·	·	·	·	·
Gray seal/ME/20220701	T	C	T	A	C	T	A	G	T	T	T	A	G	·	·	·	·	·	·	·	·	·	·	·	·
Harbor seal/ME/20220709	T	C	T	A	C	T	A	G	T	T	T	A	G	·	·	·	·	·	·	·	·	·	·	·	·
Harbor seal/ME/20220715	T	C	T	A	C	T	A	G	T	T	T	A	G	C	·	·	·	·	·	·	·	·	·	·	·
Peregrine falcon/MA/20220616	T	C	T	A	C	T	A	G	T	T	T	A	G	C	·	·	·	·	·	·	·	·	·	·	·
Common eider/MA/20220707	T	C	T	A	C	T	A	G	T	T	T	A	·	C	·	·	·	·	·	·	·	·	·	·	·
Great black-backed gull/MA/20220625	T	C	T	A	C	T	A	G	T	T	T	A	G	C	·	·	·	A	·	·	·	·	·	·	·
Harbor seal/ME/20220622	T	C	T	A	C	T	A	G	T	T	T	A	G	C	·	·	·	A	·	·	·	·	·	·	·
Harbor seal/ME/20220629	T	C	T	A	C	·	A	G	T	T	T	A	G	C	T	G	·	·	·	·	·	·	·	·	·
Harbor seal/ME/20220624	T	C	T	A	C	T	A	G	T	T	T	A	G	C	T	G	T	·	·	·	·	·	·	·	·
Peregrine falcon/MA/20220622	T	C	T	A	C	T	A	G	T	T	T	A	G	C	·	·	T	·	·	·	·	·	·	·	·
Harbor_seal/ME/20220702	T	C	T	A	C	T	A	G	T	T	T	A	G	-	T	-	T	·	·	·	·	·	·	·	·
Harbor seal/ME/20220627	T	C	T	A	C	T	A	G	T	T	T	A	G	·	·	·	·	·	·	·	C	C	G	·	·
Great black-backed gull/ME/20220707	T	C	T	A	C	T	A	G	T	T	T	A	G	·	·	·	·	·	·	·	C	C	G	C	·
Harbor seal/ME/20220628	T	C	T	A	C	T	A	G	T	T	T	A	G	·	·	·	·	·	·	·	C	C	G	C	T
Harbor seal/ME/20220626	T	C	T	A	C	T	A	G	T	T	T	A	G	·	·	·	·	·	·	·	C	C	G	C	T
Harbor seal/ME/20220702	T	C	T	A	C	T	A	G	T	T	T	A	G	·	·	·	·	·	·	G	C	C	·	·	·
Herring gull/ME/20220628	T	C	T	A	C	T	A	G	T	T	T	A	G	·	·	·	·	·	·	G	C	C	·	·	·
Common eider/ME/20220703	T	C	T	A	C	·	A	G	T	T	T	A	·	·	·	·	·	·	C	G	C	C	·	·	·
Great black-backed gull/ME/20220629	T	C	T	A	C	T	A	G	T	T	T	A	G	·	·	·	·	·	C	G	C	C	·	·	·
Herring gull/ME/20220629	T	C	T	A	C	T	A	G	T	T	T	A	G	·	·	·	·	·	·	·	·	·	·	·	·
Great black-backed gull/MA/20220708	T	C	T	A	C	T	A	G	T	T	T	A	G	·	·	·	·	·	·	·	·	·	·	·	·
Double-crested cormorant/MA/20220612	T	C	T	A	C	T	A	G	T	T	T	A	G	·	·	·	·	·	·	·	·	·	·	·	·
Canada goose/MA/20220721	T	C	T	A	C	T	A	G	T	T	T	A	T	·	·	·	·	·	·	·	·	·	·	·	·
Sanderling/MA/20220306	T	C	T	A	C	T	·	·	·	·	·	·	·	·	·	·	·	·	·	·	·	·	·	·	·
Sanderling/MA/20220301	T	C	T	A	C	·	·	·	·	·	·	·	·	·	·	·	·	·	·	·	·	·	·	·	·
Snowy owl/MA/20220313	T	C	T	·	·	·	·	·	·	·	·	·	·	·	·	·	·	·	·	·	·	·	·	·	·
Great black-backed gull/MA/20220325	T	C	T	·	·	·	·	·	·	·	·	·	·	·	·	·	·	·	·	·	·	·	·	·	·
Red-tailed hawk/MA/20220411	T	C	·	·	·	·	·	·	·	T	·	·	·	·	·	·	·	·	·	·	·	·	·	·	·
Sanderling/MA/20220302	T	C	·	·	·	·	·	·	·	·	·	·	·	·	·	·	·	·	·	·	·	·	·	·	·
Red-tailed hawk/MA/20220317	T	C	·	·	·	·	·	·	·	·	·	·	·	·	·	·	·	·	·	·	·	·	·	·	·
Red-tailed hawk/MA/20220306	·	·	·	·	·	·	·	·	·	·	·	·	·	·	·	·	·	·	·	·	·	·	·	·	·
Crow/MA/20220227	·	·	·	·	·	·	·	·	·	·	·	·	·	·	·	·	·	·	·	·	·	·	·	·	·
Crow/MA/20220321	·	·	·	·	·	·	·	G	·	·	·	·	·	·	·	·	·	·	·	·	·	·	·	·	·
Northern gannet/MA/20220510	·	·	·	·	·	·	·	·	·	·	·	·	·	·	·	·	·	·	·	·	·	·	·	·	·
Common tern/ME/20220720	·	·	·	·	·	·	·	·	·	·	·	·	·	·	·	·	·	·	·	·	·	·	·	·	·
Tern/ME/20220612	·	·	·	·	·	·	·	·	·	·	·	·	·	·	·	·	·	·	·	·	·	·	·	·	·
Harbor seal/ME/20220708	·	·	·	·	·	·	·	·	·	·	·	·	·	·	·	·	·	·	·	·	·	·	·	·	·
Harbor seal/ME/20220709	·	·	·	·	·	·	·	·	·	·	·	·	·	·	·	·	·	·	·	·	·	·	·	·	·

Primary North American lineage
2nd wave
New England-lineage a
1st wave
New England lineage b

Appendix Figure 5. Single nucleotide polymorphism pattern for New England H5N1 lineage A. Eighty-four H5N1 sequences collected from birds and seals in New England were processed by using the vSNP pipeline (https://github.com/USDA-VS/vSNP) and Chicken/NL/FAV-0033/2021 (GISAID database, https://www.gisaid.org) as a reference. Avian samples with redundant SNP motifs were removed, and the earliest observed member was retained. All single nucleotide polymorphisms (SNPs) observed in a single bird were removed. All seal SNPs were retained. Seal viruses in lineage A are highlighted in red, and seal viruses in lineage B are highlighted in blue. The SNP motif associated with New England lineage A is shown. Complete SNP data are available in Appendix 2 (https://wwwnc.cdc.gov/EID/article/29/4/22-1538-App2.xlsx).

	PB2:1824	HA:1392	NA:705	MP:681	PB1:1992	PB2:1254	PB2:1734	PB2:2097	PB1:303	PB1:1278	PB1:1497	PB1:2178	PA:681	PA:1281	PA:1971	HA:72	HA:111	HA:519	HA:1017	HA:1146	NP:66	NP:237	NA:234	NA:408	NA:663	NA:1315	NA:1317	MP:-7	NS:267	PB2:1596	PB1:639	PA:330	NS:590
Chicken/NL/FAV-0033/2021 reference	A	C	T	C	C	A	G	A	G	T	T	A	A	G	G	T	A	C	A	C	C	G	G	G	C	A	C	A	C	A	C	T	C
Peregrine falcon/MA/22MM00242/20220324	·	·	·	·	·	·	·	·	·	·	·	·	·	·	·	·	·	·	·	·	·	·	·	·	·	·	·	·	·	·	·	·	·
Great horned owl/MA/22MM00468/20220420	·	·	·	·	·	·	·	·	·	·	·	·	·	·	·	·	·	·	·	·	·	·	·	·	·	·	·	·	·	·	·	·	·
Gray seal/ME/20220701	·	·	·	·	·	·	·	·	·	·	·	·	·	·	·	·	·	·	·	·	·	·	·	·	·	·	·	·	·	·	·	·	·
Harbor seal/ME/20220709	·	·	·	·	·	·	·	·	·	·	·	·	·	·	·	·	·	·	·	·	·	·	·	·	·	·	·	·	·	·	·	·	·
Harbor seal/ME/20220715	·	·	·	·	·	·	·	·	·	·	·	·	·	·	·	·	·	·	·	·	·	·	·	·	·	·	·	·	·	·	·	·	·
Peregrine falcon/MA/20220616	·	·	·	·	·	·	·	·	·	·	·	·	·	·	·	·	·	·	·	·	·	·	·	·	·	·	·	·	·	·	·	·	·
Common eider/MA/20220707	·	·	·	·	·	·	·	·	·	·	·	·	·	·	·	·	·	·	·	·	·	·	·	·	·	·	·	·	·	·	·	·	·
Great black-backed gull/MA/20220625	·	·	·	·	·	·	·	·	·	·	·	·	·	·	·	·	·	·	·	·	·	·	·	·	·	·	·	·	·	·	·	·	·
Harbor seal/ME/20220622	·	·	·	·	·	·	·	·	·	·	·	·	·	·	·	·	·	·	·	·	·	·	·	·	·	·	·	·	·	·	·	·	·
Harbor seal/ME/20220629	·	·	·	·	·	·	·	·	·	·	·	·	·	·	·	·	·	·	·	·	·	·	·	·	·	·	·	·	·	·	·	·	·
Harbor seal/ME/20220624	·	·	·	·	·	·	·	·	·	·	·	·	·	·	·	·	·	·	·	·	·	·	·	·	·	·	·	·	·	·	·	·	·
Peregrine falcon/MA/20220622	·	·	·	·	·	·	·	·	·	·	·	·	·	·	·	·	·	·	·	·	·	·	·	·	·	·	·	·	·	·	·	·	·
Harbor_seal/ME/20220702	·	·	·	·	·	·	·	·	·	·	·	·	·	·	·	·	·	·	·	·	·	·	·	·	·	·	·	-	·	·	·	·	·
Harbor seal/ME/20220627	·	·	·	·	·	·	·	·	·	·	·	·	·	·	·	·	·	·	·	·	·	·	·	·	·	·	·	·	·	·	·	·	·
Great black-backed gull/ME/20220707	·	·	·	·	·	·	·	·	·	·	·	·	·	·	·	·	·	·	·	·	·	·	·	·	·	·	·	·	·	·	·	·	·
Harbor seal/ME/20220628	·	·	·	·	·	·	·	·	·	·	·	·	·	·	·	·	·	·	·	·	·	·	·	·	·	·	·	·	·	·	·	·	·
Harbor seal/ME/20220626	·	·	·	·	·	·	·	·	·	·	·	·	·	·	·	·	·	·	·	·	·	·	·	·	·	·	·	·	·	·	·	·	·
Harbor seal/ME/20220702	·	·	·	·	·	·	·	·	·	·	·	·	·	·	·	·	·	·	·	·	·	·	·	·	·	·	·	·	·	·	·	·	·
Herring gull/ME/20220628	·	·	·	·	·	·	·	·	·	·	·	·	·	·	·	·	·	·	·	·	·	·	·	·	·	·	·	·	·	·	·	·	·
Common eider/ME/20220703	·	·	·	·	·	·	·	·	·	·	·	·	·	·	·	·	·	·	·	·	·	·	·	·	·	·	·	·	·	·	·	·	·
Great black-backed gull/ME/20220629	·	·	·	·	·	·	·	·	·	·	·	·	·	·	·	·	·	·	·	·	·	·	·	·	·	·	·	·	·	·	·	·	·
Herring gull/ME/20220629	·	·	·	·	·	·	·	·	·	·	·	·	·	·	·	·	·	·	·	·	·	·	·	·	·	·	·	·	·	·	·	·	·
Great black-backed gull/MA/20220708	·	·	·	·	·	·	·	·	·	·	·	·	·	·	·	·	·	·	·	·	·	·	·	·	·	·	·	·	·	·	·	·	·
Double-crested cormorant/MA/20220612	·	·	·	·	·	·	·	·	·	·	·	·	·	·	·	·	·	·	·	·	·	·	·	·	·	·	·	·	·	·	·	·	·
Canada goose/MA/20220721	·	·	·	·	·	·	·	·	·	·	·	·	·	·	·	·	·	·	·	·	·	·	·	·	·	·	T	·	·	·	·	·	·
Sanderling/MA/20220306	·	·	·	·	·	·	·	·	·	·	·	·	·	·	·	·	·	·	·	·	·	·	·	·	·	·	·	·	·	·	·	·	·
Sanderling/MA/20220301	·	·	·	·	·	·	·	·	·	·	·	·	·	·	·	·	·	·	·	·	·	·	·	·	·	·	·	·	·	·	·	·	·
Snowy owl/MA/20220313	·	·	·	·	·	·	·	·	·	·	·	·	·	·	·	·	·	·	·	·	·	·	·	·	·	·	·	·	·	·	·	·	·
Great black-backed gull/MA/20220325	·	·	·	·	·	·	·	·	·	·	·	·	·	·	·	·	·	·	·	·	·	·	·	·	·	·	·	·	·	·	·	·	·
Red-tailed hawk/MA/20220411	·	·	·	·	·	·	·	·	·	·	·	·	·	·	·	·	·	·	·	·	·	·	·	·	·	·	·	·	·	·	·	·	·
Sanderling/MA/20220302	·	·	·	·	·	·	·	·	·	·	·	·	·	·	·	·	·	·	·	·	·	·	·	·	·	·	·	·	·	·	·	·	·
Red-tailed hawk/MA/20220317	·	·	·	·	·	·	·	·	·	·	·	·	·	·	·	·	·	·	·	·	·	·	·	·	·	·	·	·	·	·	·	·	·
Red-tailed hawk/MA/20220306	·	·	·	·	·	·	·	·	·	·	·	·	·	·	·	·	·	·	·	·	·	·	·	·	·	·	·	·	·	·	·	·	·
Crow/MA/20220227	·	·	·	·	·	·	·	·	·	·	·	·	·	·	·	·	·	·	·	·	·	·	·	·	·	·	·	·	·	·	·	·	·
Crow/MA/20220321	·	·	·	·	·	·	·	·	·	·	·	·	·	·	·	·	·	·	·	·	·	·	·	·	·	·	·	·	·	·	·	·	·
Northern gannet/MA/20220510	G	T	C	T	T	·	·	·	·	·	·	·	·	·	·	·	·	·	·	·	·	·	·	·	·	·	·	·	·	·	·	·	·
Common tern/ME/20220720	G	T	C	T	·	G	A	G	A	C	C	C	G	A	A	C	G	T	G	T	A	A	A	A	T	G	G	G	T	·	·	·	·
Tern/ME/20220612	G	T	C	T	T	G	A	G	A	C	C	C	G	A	A	C	G	T	G	T	A	A	A	A	T	G	G	G	T	·	·	·	·
Harbor seal/ME/20220708	G	T	C	T	T	G	A	G	A	C	C	C	G	A	A	C	G	T	G	T	A	A	A	A	T	G	G	G	T	G	A	C	A
Harbor seal/ME/20220709	G	T	C	T	T	G	A	G	A	C	C	C	G	A	A	C	G	T	G	T	A	A	A	A	T	G	G	G	T	G	A	C	A

Primary North American lineage

2nd wave

New England-lineage a

1st wave

New England lineage b

Appendix Figure 6. Single nucleotide polymorphism pattern for New England H5N1 lineage B. Eighty-four H5N1 sequences collected from birds and seals in New England were processed by using the vSNP pipeline (https://github.com/USDA-VS/vSNP) and Chicken/NL/FAV-0033/2021 (GISAID database, https://www.gisaid.org) as a reference. Avian samples with redundant SNP motifs were removed, and the earliest observed member was retained. All SNPs observed in a single bird were removed. All seal SNPs were retained. Seal viruses in lineage A are highlighted in red, and seal viruses in lineage B are highlighted in blue. The SNP motif associated with New England lineage B is shown. Complete SNP data are available in Appendix 2 (https://wwwnc.cdc.gov/EID/article/29/4/22-1538-App2.xlsx).

	PB2									
	35	531	1044	1173	1482	1596	1879	1914	2101	2112
Consensus	C	G	C	G	A	A	G	A	G	T
Gray seal/ME/22SN01540/20220701	·	·	·	·	·	·	·	·	·	C
Harbor_seal/ME/22SN01434/20220709	·	·	·	·	·	·	·	·	·	·
Harbor_seal/ME/22SN01466/20220715	·	·	·	·	·	·	·	G	·	·
Harbor seal/ME/22SN01308-9/20220622	·	·	·	·	·	·	·	·	·	·
Harbor seal/ME/22SN01393/20220629	·	·	·	·	·	·	·	·	·	·
Harbor seal/ME/22SN01328-9/20220624	·	·	·	·	·	·	·	·	·	·
Harbor seal/ME/22SN01511/20220702	·	·	·	·	·	·	·	·	·	·
Harbor seal/ME/22SN01358-60/20220627	T	·	·	·	·	·	·	·	A	·
Harbor seal/ME/22SN01376/20220628	·	A	·	A	C	·	A	·	·	·
Harbor seal/ME/22SN01339-42/20220626	·	·	T	·	C	·	·	·	·	·
Harbor seal/ME/22SN01522/20220702	·	·	·	·	·	·	·	·	·	·
Harbor_seal/ME/22SN01410/20220708	·	·	·	·	·	G	·	·	·	·
Harbor_seal/ME/22SN01431/20220709	·	·	·	·	·	G	·	·	A	·

	PB1												
	37	261	632	639	813	894	1266	1329	1479	1509	1567	1579	2127
Consensus	C	C	G	C	C	C	A	C	A	T	A	G	T
Gray seal/ME/22SN01540/20220701	·	·	·	·	·	·	·	·	·	·	·	·	·
Harbor_seal/ME/22SN01434/20220709	·	·	A	·	·	·	G	·	·	·	·	·	·
Harbor_seal/ME/22SN01466/20220715	·	·	·	·	·	·	·	·	·	·	C	A	·
Harbor seal/ME/22SN01308-9/20220622	·	·	·	·	·	·	·	·	·	·	·	·	·
Harbor seal/ME/22SN01393/20220629	T	·	·	·	·	·	·	·	·	·	·	·	·
Harbor seal/ME/22SN01328-9/20220624	T	·	·	·	·	·	·	·	·	·	·	·	·
Harbor seal/ME/22SN01511/20220702	T	·	·	·	·	A	·	·	G	·	·	·	·
Harbor seal/ME/22SN01358-60/20220627	·	·	·	·	·	·	·	·	·	C	·	·	·
Harbor seal/ME/22SN01376/20220628	·	T	·	·	·	·	·	·	·	·	·	·	·
Harbor seal/ME/22SN01339-42/20220626	·	T	·	·	·	·	·	·	·	·	·	·	·
Harbor seal/ME/22SN01522/20220702	·	·	·	·	·	·	·	A	·	·	·	·	C
Harbor_seal/ME/22SN01410/20220708	·	·	·	A	·	·	·	·	·	·	·	·	·
Harbor_seal/ME/22SN01431/20220709	·	·	·	A	A	·	·	·	·	·	·	·	·

	PA														
	78	112	291	302	330	420	755	788	1135	1273	1293	1353	1371	1470	1524
Consensus	A	A	C	A	T	A	A	C	G	C	C	C	A	A	A
Gray seal/ME/22SN01540/20220701	·	·	·	·	·	C	·	·	A	·	·	·	G	·	G
Harbor_seal/ME/22SN01434/20220709	·	·	·	G	·	·	G	·	·	·	·	·	·	·	·
Harbor_seal/ME/22SN01466/20220715	·	·	·	·	·	·	·	·	·	·	·	·	·	·	·
Harbor seal/ME/22SN01308-9/20220622	·	·	·	·	·	·	·	A	·	T	·	·	·	·	·
Harbor seal/ME/22SN01393/20220629	·	·	·	·	·	·	·	·	·	·	·	T	·	·	·
Harbor seal/ME/22SN01328-9/20220624	·	G	·	·	·	·	·	·	·	·	T	·	·	G	·
Harbor seal/ME/22SN01511/20220702	·	G	·	·	·	·	·	·	·	·	T	·	·	·	·
Harbor seal/ME/22SN01358-60/20220627	G	·	·	·	·	·	·	·	·	·	·	·	·	·	·
Harbor seal/ME/22SN01376/20220628	G	·	·	·	·	·	·	·	·	·	·	·	·	·	·
Harbor seal/ME/22SN01339-42/20220626	G	·	·	·	·	·	·	·	·	·	·	·	·	·	·
Harbor seal/ME/22SN01522/20220702	·	·	T	·	·	·	·	·	·	·	·	·	·	·	·
Harbor_seal/ME/22SN01410/20220708	·	·	·	·	C	·	·	·	·	·	·	·	·	·	·
Harbor_seal/ME/22SN01431/20220709	·	·	·	·	C	·	·	·	·	·	·	·	·	·	·

	HA						
	180	408	601	1130	1140	1605	1612
Consensus	A	T	G	G	T	T	G
Gray seal/ME/22SN01540/20220701	·	·	·	·	·	·	·
Harbor_seal/ME/22SN01434/20220709	·	·	·	·	·	·	T
Harbor_seal/ME/22SN01466/20220715	·	·	A	A	·	C	·
Harbor seal/ME/22SN01308-9/20220622	G	·	·	·	·	·	·
Harbor seal/ME/22SN01393/20220629	·	·	·	·	·	·	·
Harbor seal/ME/22SN01328-9/20220624	·	·	·	·	·	·	·
Harbor seal/ME/22SN01511/20220702	·	·	·	·	·	·	·
Harbor seal/ME/22SN01358-60/20220627	·	C	·	·	·	·	·
Harbor seal/ME/22SN01376/20220628	·	·	·	·	·	·	·
Harbor seal/ME/22SN01339-42/20220626	·	·	·	·	·	·	·
Harbor seal/ME/22SN01522/20220702	·	·	·	·	·	·	·
Harbor_seal/ME/22SN01410/20220708	·	·	·	·	·	·	·
Harbor_seal/ME/22SN01431/20220709	·	·	·	·	A	·	·

	NP														
	234	342	493	541	843	936	953	1072	1164	1227	1244	1255	1342	1363	1453
Consensus	T	G	T	G	C	C	C	C	A	G	A	C	A	G	G
Gray seal/ME/22SN01540/20220701	·	A	·	·	·	A	·	·	·	·	·	·	G	A	·
Harbor_seal/ME/22SN01434/20220709	C	·	·	·	T	·	·	·	·	·	·	·	·	·	·
Harbor_seal/ME/22SN01466/20220715	·	·	·	·	·	·	·	T	·	·	·	·	G	·	·
Harbor seal/ME/22SN01308-9/20220622	·	·	C	·	·	·	·	·	·	·	·	·	·	·	·
Harbor seal/ME/22SN01393/20220629	·	·	·	·	·	·	·	·	·	·	·	·	·	·	·
Harbor seal/ME/22SN01328-9/20220624	·	·	·	·	·	·	·	·	C	·	·	·	·	·	·
Harbor seal/ME/22SN01511/20220702	·	·	·	·	·	·	·	·	C	·	G	·	·	·	·
Harbor seal/ME/22SN01358-60/20220627	·	·	·	·	·	·	·	·	·	·	·	·	·	·	·
Harbor seal/ME/22SN01376/20220628	·	·	·	·	·	·	A	·	·	·	·	·	·	·	·
Harbor seal/ME/22SN01339-42/20220626	·	·	·	·	·	·	·	·	·	·	·	·	·	·	A
Harbor seal/ME/22SN01522/20220702	·	·	·	·	·	·	·	·	·	G	·	T	·	·	·
Harbor_seal/ME/22SN01410/20220708	·	·	·	T	·	·	·	·	·	C	·	·	·	·	·
Harbor_seal/ME/22SN01431/20220709	·	·	·	·	·	·	·	·	·	·	·	·	·	·	·

	NA						MP
	69	156	507	933	1027	1406	326
Consensus	G	C	G	G	G	A	T
Gray seal/ME/22SN01540/20220701	A	·	·	A	·	·	·
Harbor_seal/ME/22SN01434/20220709	·	·	·	·	·	G	·
Harbor_seal/ME/22SN01466/20220715	·	·	·	·	·	·	·
Harbor seal/ME/22SN01308-9/20220622	·	·	·	·	·	·	·
Harbor seal/ME/22SN01393/20220629	·	·	·	·	·	·	·
Harbor seal/ME/22SN01328-9/20220624	·	·	·	·	·	·	·
Harbor seal/ME/22SN01511/20220702	·	·	·	·	·	·	·
Harbor seal/ME/22SN01358-60/20220627	·	·	·	·	·	·	·
Harbor seal/ME/22SN01376/20220628	·	·	·	·	·	·	C
Harbor seal/ME/22SN01339-42/20220626	·	·	·	·	·	·	·
Harbor seal/ME/22SN01522/20220702	·	·	A	·	A	·	·
Harbor_seal/ME/22SN01410/20220708	·	T	·	·	·	·	·
Harbor_seal/ME/22SN01431/20220709	·	·	·	·	·	·	·

	NS									
	-1	111	259	294	354	590	726	737	739	784
Consensus	A	C	T	G	A	C	A	A	T	A
Gray seal/ME/22SN01540/20220701	·	·	A	·	·	·	·	·	·	·
Harbor_seal/ME/22SN01434/20220709	·	·	·	·	·	·	·	G	C	·
Harbor_seal/ME/22SN01466/20220715	·	·	·	·	·	·	·	·	·	·
Harbor seal/ME/22SN01308-9/20220622	·	·	·	A	·	·	·	·	·	·
Harbor seal/ME/22SN01393/20220629	G	A	·	·	·	·	·	·	·	·
Harbor seal/ME/22SN01328-9/20220624	G	·	·	·	·	·	·	·	·	·
Harbor seal/ME/22SN01511/20220702	-	·	·	·	·	·	·	·	·	G
Harbor seal/ME/22SN01358-60/20220627	·	·	·	·	·	·	G	·	·	·
Harbor seal/ME/22SN01376/20220628	·	·	·	·	·	·	·	·	·	·
Harbor seal/ME/22SN01339-42/20220626	·	·	·	·	·	·	·	·	·	·
Harbor seal/ME/22SN01522/20220702	·	·	·	·	G	·	·	·	·	·
Harbor_seal/ME/22SN01410/20220708	·	·	·	·	·	A	·	·	·	·
Harbor_seal/ME/22SN01431/20220709	·	·	·	·	·	A	·	·	·	·

Appendix Figure 7. Single nucleotide polymorphisms observed in seal-derived H5N1 viruses. Thirteen H5N1 sequences collected from seals in New England were processed by using the vSNP pipeline (https://github.com/USDA-VS/vSNP) and Chicken/NL/FAV-0033/2021 (GISAID database, https://www.gisaid.org) as a reference. All SNPs observed in each seal are shown. Seal viruses in lineage A are highlighted in red, and seal viruses falling into lineage B are highlighted in blue. Complete SNP data are available in Appendix 2 (https://wwwnc.cdc.gov/EID/article/29/4/22-1538-App2.xlsx).
